# Unveiling the potential geographic range of *Lepidium meyenii* (Maca) and its climate-driven shifts in a changing climate

**DOI:** 10.3389/fpls.2026.1839669

**Published:** 2026-07-15

**Authors:** Zeyu Qin, Huasheng Huang, Xuanqi Liu, Xia Meng, Minqiao Li

**Affiliations:** 1 School of Geography and Planning, Sun Yat-sen University, Guangzhou, China; 2 Carbon-Water Observation and Research Station in Karst Regions of Northern Guangdong, School of Geography and Planning, Sun Yat-sen University, Guangzhou, China

**Keywords:** biogeography, Brassicaceae, climate change, ecological niche model, *Lepidium meyenii*, species distribution

## Abstract

Global warming has induced pronounced phenological shifts and community degradation in alpine herbaceous plants. However, their spatiotemporal distributions, future responses to climate change, and the underlying environmental drivers remain poorly understood. Here, we focus on *Lepidium meyenii*, a medicinal herb native to the high-altitude regions of the Andes Mountains in South America, which may face habitat contraction and potential population decline in the future due to changing environments. We integrate species distribution and environmental data with a random forest model to project the potential suitable habitats of *L. meyenii* across time and identify their key environmental drivers. We find that the most significant environmental variables that impact the distribution of *L. meyenii* include elevation, temperature annual range (bio7), and mean diurnal range (bio2). The species generally favors high-elevation environments characterized by high bio2 and low bio7 values. The Andes Mountains have consistently remained a stably suitable habitat throughout the study period, whereas climate change has reshaped the potential suitability patterns in other regions. From the Last Glacial Maximum to the present, the potential suitable habitat for *L. meyenii* shows a declining trend, with the most pronounced contraction occurring on the Tibetan Plateau. However, under future climate scenarios, this trend is projected to shift. With increasing emission scenarios, environmental suitability in the southern Tibetan Plateau is expected to increase, and potential suitable habitat is projected to expand, suggesting that this region could become an important area for the cultivation and sustainable utilization of *L. meyenii*. This study provides valuable insights into the distribution patterns of alpine herbaceous plants in response to climate change and contributes to biodiversity conservation efforts and the sustainable management of medicinal plants as biological resources.

## 1 Introduction

Climate change alters long-term climate patterns, exacerbates the frequency and severity of extreme climatic events (e.g., heavy rainstorms, droughts, and storms) (Stott, 2016; Intergovernmental Panel on Climate Change (IPCC), 2021), and is also increasingly recognized as a key driver of species distribution shifts (Wang et al., 2024; Zhao et al., 2021). Many species are moving to higher altitudes and latitudes as temperatures rise (Kelly and Goulden, 2008; Lu, 2025). These niche shifts occur at a much slower rate than climate change (Jezkova and Wiens, 2016). As a result, species may struggle to adapt to rapidly changing environments, thereby increasing the potential risk of species extinction and biodiversity loss. Therefore, it is essential to understand the relationship between climate change and species distribution for developing effective conservation strategies, strengthening ecosystem management, and supporting long-term ecological planning.

Species distribution models (SDMs) project potential suitable habitats of species by analyzing correlations between species occurrence records and environmental variables, and are widely used to assess species distributions under past, current, and future climate scenarios and to explore the potential impacts of climate change (Liu et al., 2025; Miller, 2010; Tang et al., 2025). However, differences among SDMs in model structure, parameterization, and the formulation of species-environment relationships may lead to substantial variation in model performance and prediction, thereby introducing uncertainty into species distribution projections (Hao et al., 2020). Model selection and parameter optimization are therefore essential for improving predictive accuracy and reliability (Muscarella et al., 2014). As an integrated modeling platform, “biomod2” incorporates a suite of widely used SDMs and provides tools for model evaluation, parameter customization, and ensemble forecasting (Thuiller et al., 2016; Wang et al., 2025). By facilitating comparisons among alternative models and identifying the best-performing models, “biomod2” provides an effective framework for assessing model uncertainty and improving the robustness of species distribution projections. Consequently, it has been widely applied in SDMs (Wang et al., 2025).

Alpine ecosystems are widely regarded as among the most climate-sensitive biomes (Walther et al., 2005). Steep elevational gradients and complex topography generate pronounced environmental heterogeneity (Pérez-Giraldo et al., 2026). Alpine plants typically possess narrow ecological niches, high habitat specialization, and limited dispersal capacity; these render them particularly vulnerable to climatic changes (Timoshok et al., 2016). Past climate fluctuations have profoundly shaped the historical distribution patterns of alpine plants and the formation of glacial refugia (Schorr et al., 2012). Ongoing global warming is transforming alpine ecosystems by accelerating gravelization and meadow degradation and by altering the phenology, growth, and population dynamics of alpine species (Cui et al., 2022; Sun et al., 2021). Emerging evidence suggests that future climate change will further shift suitable habitat, distribution centers, and elevational ranges of alpine plants, increasing the risk of local extirpation for some taxa (e.g., Adde et al., 2024). Assessing alpine plant responses to climate change and projecting potential distribution dynamics are therefore essential for understanding biodiversity trajectories in mountain ecosystems, identifying future refugia, and informing conservation and management strategies.

*Lepidium meyenii* Walp. belongs to the genus *Lepidium* L. and is an alpine herbaceous species native to the Andes of western South America (https://powo.science.kew.org). It diverged in the middle Miocene (~12 Ma), and a whole-genome duplication event (~6.7 Ma) promoted the functional diversification of adaptive genes, shaping leaf morphology and self-fertility traits suited to alpine environments (Zhang et al., 2016). It is commonly referred to as “Peruvian ginseng” and possesses significant economic value due to its rich nutrient profile and numerous health benefits (Gonzales et al., 2004; Rubio et al., 2006; Da Silva Leitáo Peres et al., 2020). Consequently, products derived from *L. meyenii* have gained considerable commercial popularity (Yin et al., 2019). Since the 1990s, cultivation attempts in the United States, Japan, and Germany have met with limited success, while China successfully domesticated *L. meyenii* in 2002 and has established a substantial commercial market. For example, in Yunnan Province, the cultivation area of *L. meyenii* has reached 22, 000 hm^2^ (He et al., 2016; Chen et al., 2021).

Previous studies on *L. meyenii* have largely focused on its nutritional composition, medicinal properties, and phylogeny (e.g., Gonzales et al., 2004; Rubio et al., 2006; Zhang et al., 2016). In contrast, its geographic distribution patterns, key environmental determinants, and responses to climate change, across past, present, and future time periods remain poorly understood. Given its adaptation to alpine environments and the growing extent of cultivation outside its native range, *L. meyenii* is likely to be highly sensitive to climatic shifts that may alter its potential distribution. It is therefore important to clarify the key environmental factors and spatiotemporal dynamics of its suitable habitat under different climate scenarios.

To address these issues, we employed “biomod2” to compare the predictive performance of multiple SDMs and select the best-performing algorithm. Based on this model, we projected the potential distribution of *L. meyenii* under past, present, and future climatic conditions. Specifically, we asked: 1) Which environmental variables most strongly influence the distribution of *L. meyenii*? 2) How has its suitable habitat shifted from the past to the present? and 3) How is future climate change likely to modify the spatial patterns of habitat suitability? By addressing these questions, we provide new insights into the climatic responses and environmental adaptation of *L. meyenii*, and offer a scientific basis for its conservation, introduction, cultivation, and sustainable use, as well as for the management of other alpine herbaceous species.

## 2 Data and methods

### 2.1 The natural distribution of *L. meyenii* and the study area

**Figure 1:**
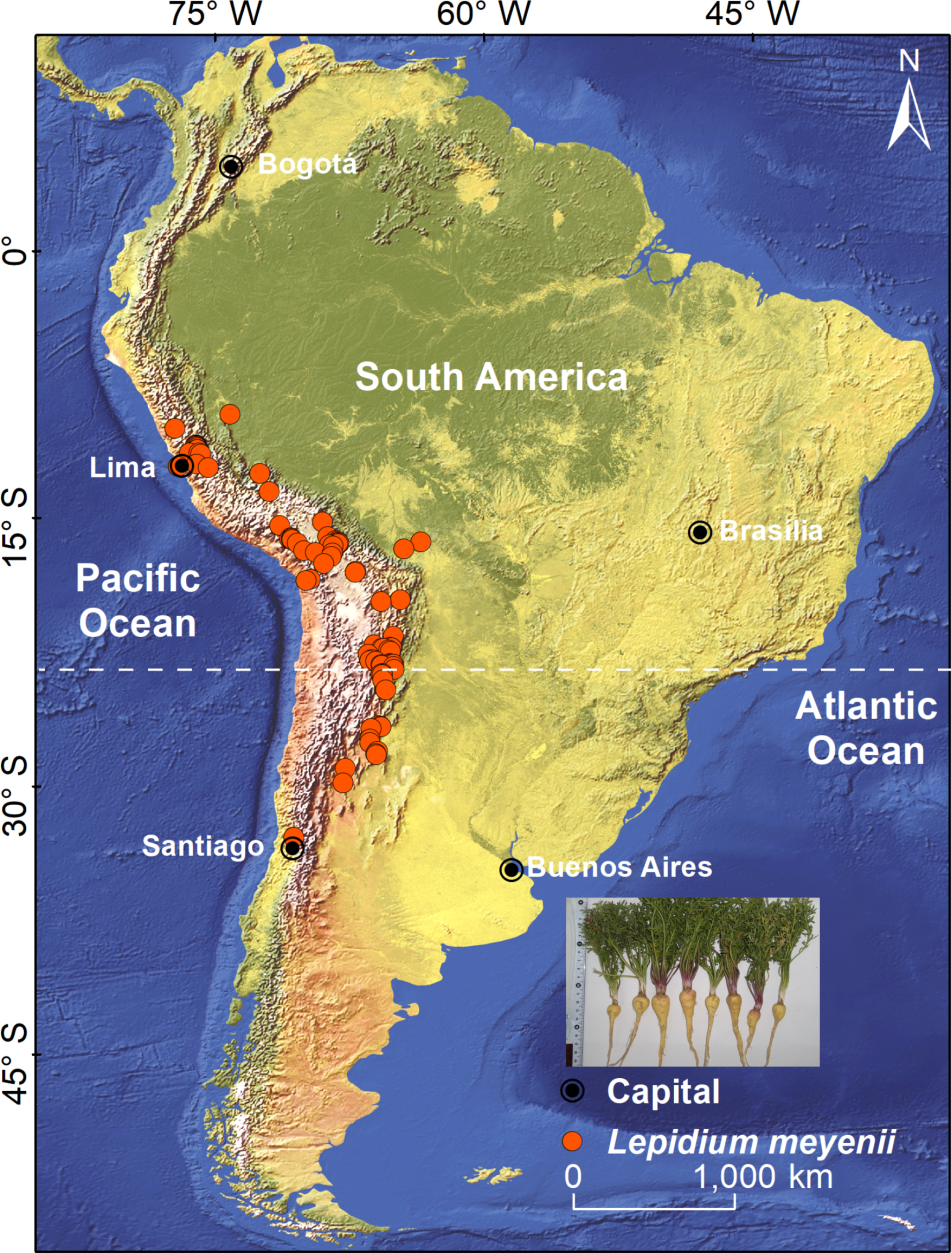
Geographically valid occurrence records of *Lepidium meyenii* sourced from GBIF (http://www.gbif.org). The base map and the embedded image of *L. meyenii* were obtained from Natural Earth (https://www.naturalearthdata.com/) and Wikipedia (https://en.wikipedia.org/, credit: Vahe Martirosyan, under the CC BY 2.0 license, https://creativecommons.org/licenses/by/2.0/), respectively.

*L. meyenii* is primarily distributed along the western coast of South America within the Andes, encompassing parts of northwestern Argentina, Bolivia, Peru, and the western region of Brazil (**Figure 1**). The Andes Mountain Range stretches from north to south, with its average elevation decreasing and then increasing along its length. Within the latitudinal range of 10–30° S, the average altitude is ca. 3000 m, which coincides with the area where *L. meyenii* is most densely populated (Montgomery et al., 2001). The climate in western South America is highly complex, shaped by the perennial effects of the Peru Current, the barrier created by the Andes, and the South American monsoon system. These factors contribute to a diverse range of climates from north to south, including tropical arid, tropical humid, savanna, semi-arid, and temperate oceanic climates (Garreaud, 2009; Toledo et al., 2022).

Owing to its significant nutritional, medicinal, and high economic value, *L. meyenii* has been introduced for cultivation outside its native range, particularly in Yunnan Province in southwest China emerging as one of the major cultivation areas worldwide (Gonzales et al., 2004; Rubio et al., 2006; He et al., 2016; Chen et al., 2021). Given the dual importance of protecting wild resources in its native range and guiding scientific agricultural cultivation in introduced regions, it is insufficient to limit the study area to South America. Moreover, many studies have also provided examples of global potential distribution projections, which serve as a reference for this study (Yoon et al., 2026). Therefore, we aim to project the global potential suitable habitats of *L. meyenii* in past, present, and future climate scenarios.

### 2.2 The species distribution data collection and screening

The occurrence records of *L. meyenii* were compiled from the Global Biodiversity Information Facility (https://www.gbif.org; GBIF.org, 2026; n = 495), iNaturalist (https://www.inaturalist.org; iNaturalist, 2026; n = 19), and the Botanical Information and Ecology Network database (Maitner et al., 2018; n = 82). All records were subsequently subjected to a systematic data-cleaning procedure. We first used ENMTools to eliminate duplicate coordinates and performed random gridding, ensuring that only one occurrence per 2.5 arc-minutes grid cell was retained. We then applied the “clean_coordinates” function from the “CoordinateCleaner” R package v3.0.1 (Zizka et al., 2019) to exclude records with potentially erroneous geographical coordinates. Based on previous studies and the Plants of the World Online (https://www.powo.science.kew.org), we confirmed that *L. meyenii* is native only to northwestern Argentina, Bolivia, northern Chile, and Peru (Dini et al., 1994). Therefore, all occurrence records outside these regions (e.g., Yunnan, China) were excluded. These records do not represent the natural distribution of the species and may therefore violate the fundamental assumption of ecological niche modeling, potentially obscuring the true climatic niche of the species and introducing anthropogenic bias into the model. Finally, we obtained 86 valid natural occurrence records, which exceed the sample size requirements commonly reported for species distribution modeling, offering a rational foundation for modeling (Van Proosdij et al., 2016).

### 2.3 Climate data, and environmental variables

We selected 22 environmental variables from the WorldClim database (http://worldclim.org), including 19 bioclimatic and 3 topographic variables (**Supplementary Material**, **Supplementary Table 1**). To ensure spatial consistency across all periods, all environmental variables were selected at a spatial resolution of 2.5 arc-minutes from the WorldClim database and maintained at this resolution throughout all projections, which is suitable for global-scale modeling of potential suitable habitats. The present-day climate data are WorldClim version 2.1 climate data for 1970–2000 (Fick and Hijmans, 2017).

For paleoclimate projections, we projected the potential suitable habitats of *L. meyenii* for the Last Glacial Maximum (LGM, ca. 22, 000 years ago) and the Mid-Holocene (MH, ca. 6, 000 years ago) using downscaled CMIP5 climate data (calibrated and bias-corrected with WorldClim v1.4 as the baseline “current” climate). For the LGM, we used climate data from three CMIP5 global climate models (GCMs) (CCSM4, MIROC-ESM, and MPI-ESM-P). For the MH, we used nine CMIP5 GCMs (BCC-CSM1-1, CCSM4, CNRM-CM5, HadGEM2-CC, HadGEM2-ES, IPSL-CM5A-LR, MIROC-ESM, MPI-ESM-P, and MRI-CGCM3). We then averaged multiple models to smooth out differences and reduce the bias of individual models.

For future projections, we focused on two periods, the 2050s (averages for 2041–2060) and the 2090s (averages for 2081–2100), to reflect short-term and long-term trends in potential suitable habitat changes. We derived these future data from downscaled CMIP6 climate projections (calibrated and bias-corrected with WorldClim v2.1 as the baseline climate). We also averaged the outputs from three CMIP6 GCMs (FIO-ESM-2-0, MPI-ESM1-2-HR, and MRI-ESM2-0), which have been reported to perform relatively well in South America and East Asia, to reduce inter-model variability and minimize individual model bias (Zhang et al., 2024). For each future period, we applied two climate scenarios from the CMIP6 framework: SSP245 (a moderate-emission scenario) and SSP585 (a high-emission and pessimistic scenario) (O’Neill et al., 2016).

To reduce the effects of multicollinearity among environmental variables on model performance and to avoid model overfitting, we conducted Pearson and Spearman correlation analyses using R. Variables showing high pairwise correlation (|r| ≥ 0.8) were excluded. Ultimately, nine environmental variables were retained for model simulations, including annual mean temperature (bio1), mean diurnal range (bio2), temperature annual range (bio7), annual precipitation (bio12), precipitation of driest month (bio14), precipitation seasonality (bio15), aspect, slope, and elevation (**Supplementary Material**, **Supplementary Figures 1**, **2**). To further evaluate multicollinearity among the selected predictors, we additionally conducted variance inflation factor (VIF) analysis, which is commonly used to detect redundancy and collinearity among environmental variables (Naimi et al., 2025). The results showed that all retained variables had acceptable VIF values (< 10) (**Supplementary Material**, **Supplementary Table 2**). Subsequently, “biomod2” was used to assess the relative contribution of each predictor to model performance, and variables with a relative contribution of less than 1% are typically excluded (Thuiller et al., 2016). The results indicated that bio12, bio14, and aspect had relative contributions below 1%, and were therefore excluded from subsequent analyses.

### 2.4 Model selection

We conducted model analysis using the R packages “ENMeval”, ”dismo” and “biomod2” (Muscarella et al., 2014; Thuiller et al., 2016; Hijmans et al., 2017). All environmental variables were first converted to ASCII format in ArcGIS 10.8.1. Before modelling, these variables were processed and standardized with “dismo”, including raster alignment, extent matching, and resolution harmonization, to ensure consistency across all predictor layers (Hijmans et al., 2017). The “ENMeval” was utilized to optimize MaxEnt model parameters (Elith et al., 2011; Muscarella et al., 2014), enabling us to test various feature combinations (FC) and regularization multiplier (RM) to identify the most suitable settings. The MaxEnt software offers five types of FC: L (linear), Q (quadratic), H (hinge), P (product), and T (threshold). To refine the model, we tested RM values from 0.5 to 6 in increments of 0.5, resulting in a total of 12 RM values. Additionally, we evaluated six different FC combinations: L, LQ, H, LQH, LQHP, and LQHPT. In total, we assessed 72 parameter combinations using the corrected Akaike Information Criterion (AICc), selecting the configuration with the lowest AICc value to ensure an optimal balance between model fit and complexity. The optimal model had feature classes FC = LQ and RM = 1, with the lowest AICc value of 1818.93 (**Supplementary Material**, **Supplementary Table 3**).

Considering that MaxEnt models optimized using “ENMeval” have been widely applied in SDMs, we further compared this approach with the other models in “biomod2” to evaluate the predictive performance of mainstream models and identify the best-performing model for simulating potential suitable habitats (Tang et al., 2025; Liu et al., 2025).

We subsequently used “biomod2” to construct models with 11 algorithms: the maximum entropy model (MaxEnt), random forest (RF), generalized additive model (GAM), generalized linear model (GLM), artificial neural network (ANN), classification tree analysis (CTA), flexible discriminant analysis (FDA), generalized boosting model (GBM), multivariate adaptive regression splines (MARS), surface range envelope (SRE), and extreme gradient boosting (XGBoost). In “biomod2”, two MaxEnt models were included: one using the ENMeval-optimized parameter setting from the previous step, and another using the “BigBoss” parameter settings integrated in “biomod2”, which provide empirically optimized configurations for every model. The other 10 models were similarly configured with the “BigBoss” parameter settings. To improve model robustness, the dataset was randomly partitioned into a training set (75%) and a testing set (25%), with each model repeated 20 times. We also generated 260 pseudo-absence points, corresponding three times the number of occurrence records recommended by Barbet‐Massin et al. (2012).

Finally, the selection of the best-performing model was based on the receiver operating characteristic (ROC) and true skill statistics (TSS) (Allouche et al., 2006; Lobo et al., 2008). We analyzed the area under the curve (AUC) and TSS values to compare the performance of 11 models in simulating the potential suitable habitats for *L. meyenii*. Model performance was evaluated with the following criteria: (1) poor (AUC < 0.6), (2) fair (0.6 ≤ AUC < 0.8), (3) good (0.8 ≤ AUC < 0.9), and (4) excellent (0.9 ≤ AUC < 1) (Lobo et al., 2008). Additionally, a TSS value greater than 0.8 is generally considered indicative of excellent model performance (Allouche et al., 2006).

### 2.5 Classification of habitat suitability and centroid analysis of suitable areas

To reduce subjectivity in habitat suitability classification, we referred to previously published SDM studies and classified habitat suitability into four categories in ArcGIS 10.8.1: 0–0.2 (unsuitable), 0.2–0.4 (lowly suitable), 0.4–0.6 (moderately suitable), and 0.6–1 (highly suitable) (Wang et al., 2024). These categories were subsequently reclassified as follows: 0–0.2 = 0, 0.2–0.4 = 1, 0.4–0.6 = 2, and 0.6–1 = 3. Raster calculations were performed as the difference between suitability maps of later and earlier periods (later minus earlier). In this context, values < 0, = 0, and > 0 indicate potential suitable habitats of contraction, unchanged, and expansion, respectively. Compared with directly subtracting continuous raster layers from different time periods, this approach reduces noise caused by minor fluctuations and emphasizes changes among distinct habitat suitability levels.

Using the centroid calculation function in ArcGIS 10.8.1, we also calculated the centroids of moderately and highly suitable habitats for *L. meyenii* across three time periods: the past, present and future in both South America and East Asia. This analysis allows for the comparison of differences in the spatial distribution centers of potential suitable habitats for *L. meyenii* among different climatic scenarios.

## 3 Results

### 3.1 The random forest has the optimal performance

**Figure 2:**
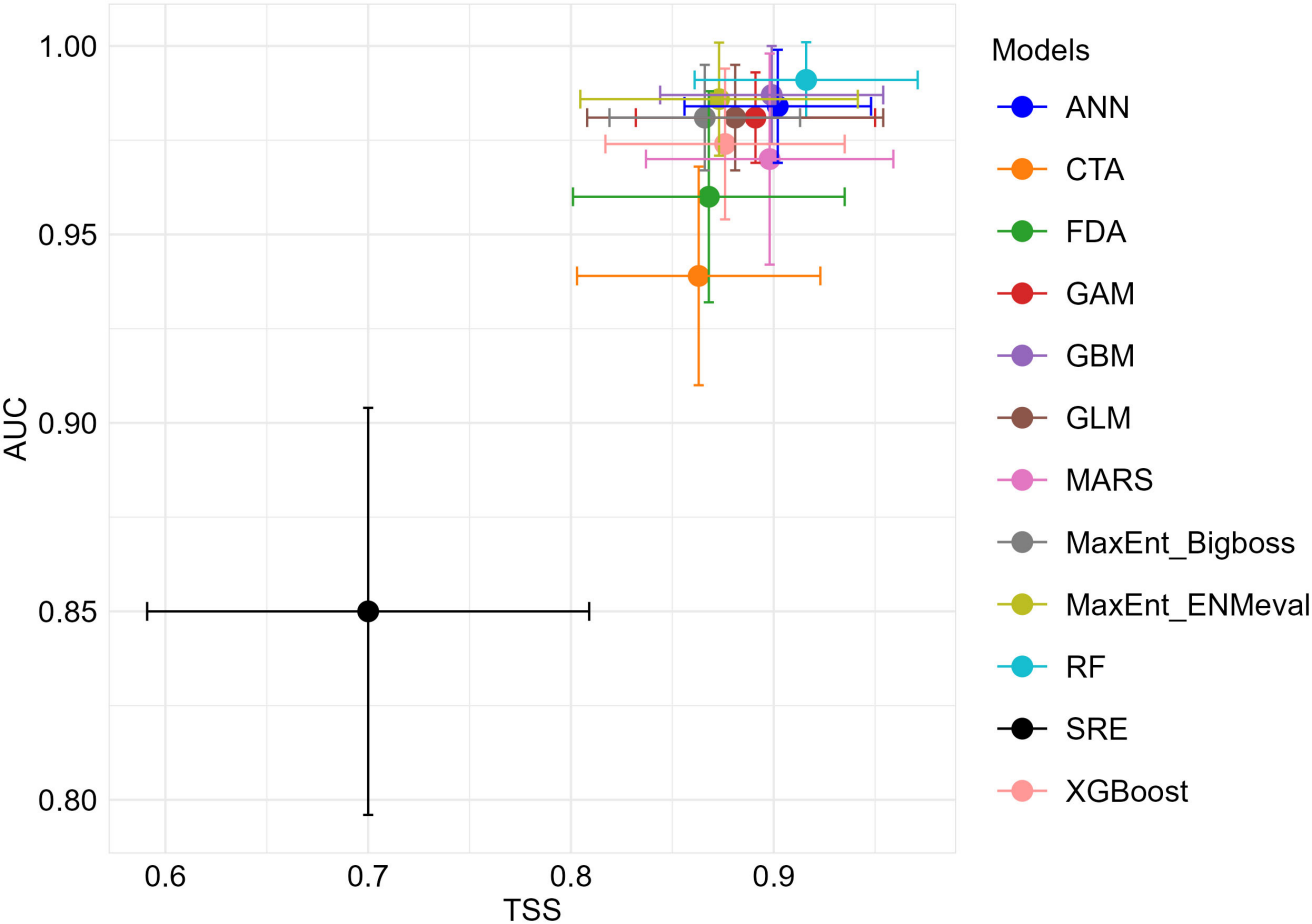
Evaluation of the accuracy of 11 models for simulating the global potential distribution for *Lepidium meyenii* using the biomod2 package. The error bars represent the standard deviation of area under the curve (AUC) and true skill statistic (TSS). The models evaluated include artificial neural network (ANN), classification tree analysis (CTA), flexible discriminant analysis (FDA), generalized additive model (GAM), generalized boosting model (GBM), generalized linear model (GLM), multiple adaptive regression splines (MARS), maximum entropy model with “BigBoss” parameter settings (MaxEnt_Bigboss), maximum entropy model with ENMeval-optimized parameter settings (MaxEnt_ENMeval), random forest (RF), surface range envelope (SRE), and extreme gradient boosting (XGBoost). Except for MaxEnt_ENMeval, which used parameter settings optimized by the ENMeval package, all other models were implemented using the “BigBoss” parameter settings integrated in biomod2.

Based on AUC and TSS values, and the actual simulation outcomes, the RF model shows the best performance, with an AUC value of 0.991 and a TSS value of 0.916 (**Figure 2**). These metrics demonstrate its superior accuracy in predicting the distribution of suitable habitats for *L. meyenii*.

### 3.2 Elevation and temperature annual range (bio7) are the most critical factors

**Figure 3:**
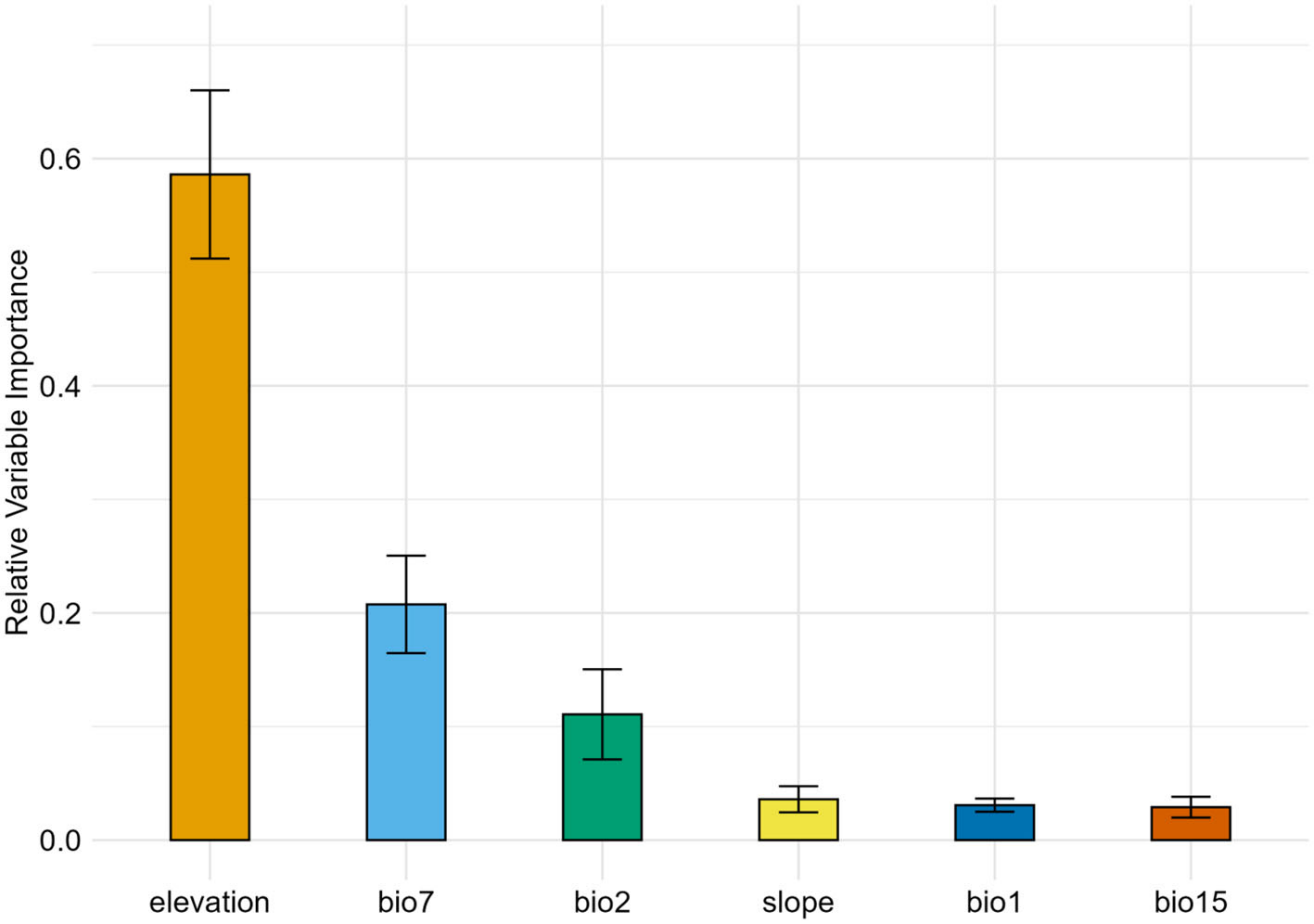
Relative importance of environmental variables on the current potential distribution of *Lepidium meyenii*. The error bars represent the standard deviation of relative importance for each environmental variable. These variables include annual mean temperature (bio1), mean diurnal range (bio2), temperature annual range (bio7), precipitation seasonality (bio15), slope, and elevation.

The present-day distribution of *L. meyenii* is shaped by both climatic and topographic factors (**Figure 3**). The most significant environmental variables influencing this distribution are elevation (58.6%), temperature annual range (bio7) (20.7%), mean diurnal range (bio2) (11.1%), slope (3.6%), annual mean temperature (bio1) (3.1%), precipitation seasonality (bio15) (2.9%) (**Figure 3**). Consequently, elevation and the bio7 emerge as the most critical factors that influence the current distribution of *L. meyenii*.

**Figure 4:**
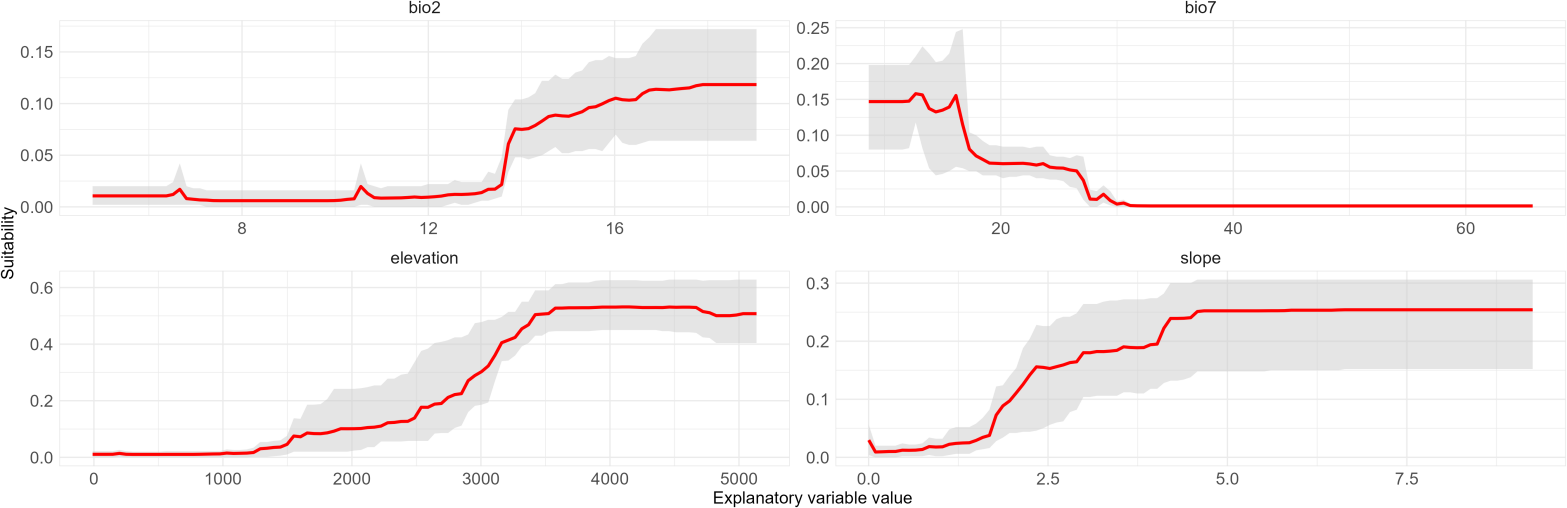
Response curves of the distribution of *Lepidium meyenii* in relation to the four most important environmental variables. They are elevation (meters), mean diurnal range (bio2) (°C), temperature annual range (bio7) (°C) and slope (°). The grey background indicates the fluctuations observed across multiple random simulations.

The habitat suitability for *L. meyenii* shows a positive correlation with elevation, bio2, and slope. Specifically, *L. meyenii* thrives at elevations above 2000 m, where the mean diurnal range exceeds 14 °C, and slopes are greater than 2.5° (**Figure 4**). Conversely, the habitat suitability is negatively correlated with bio7. This indicates that *L. meyenii* grows better when temperature annual range is less than 20 °C.

### 3.3 Potential suitable habitats under past, current, and future climate conditions

**Figure 5:**
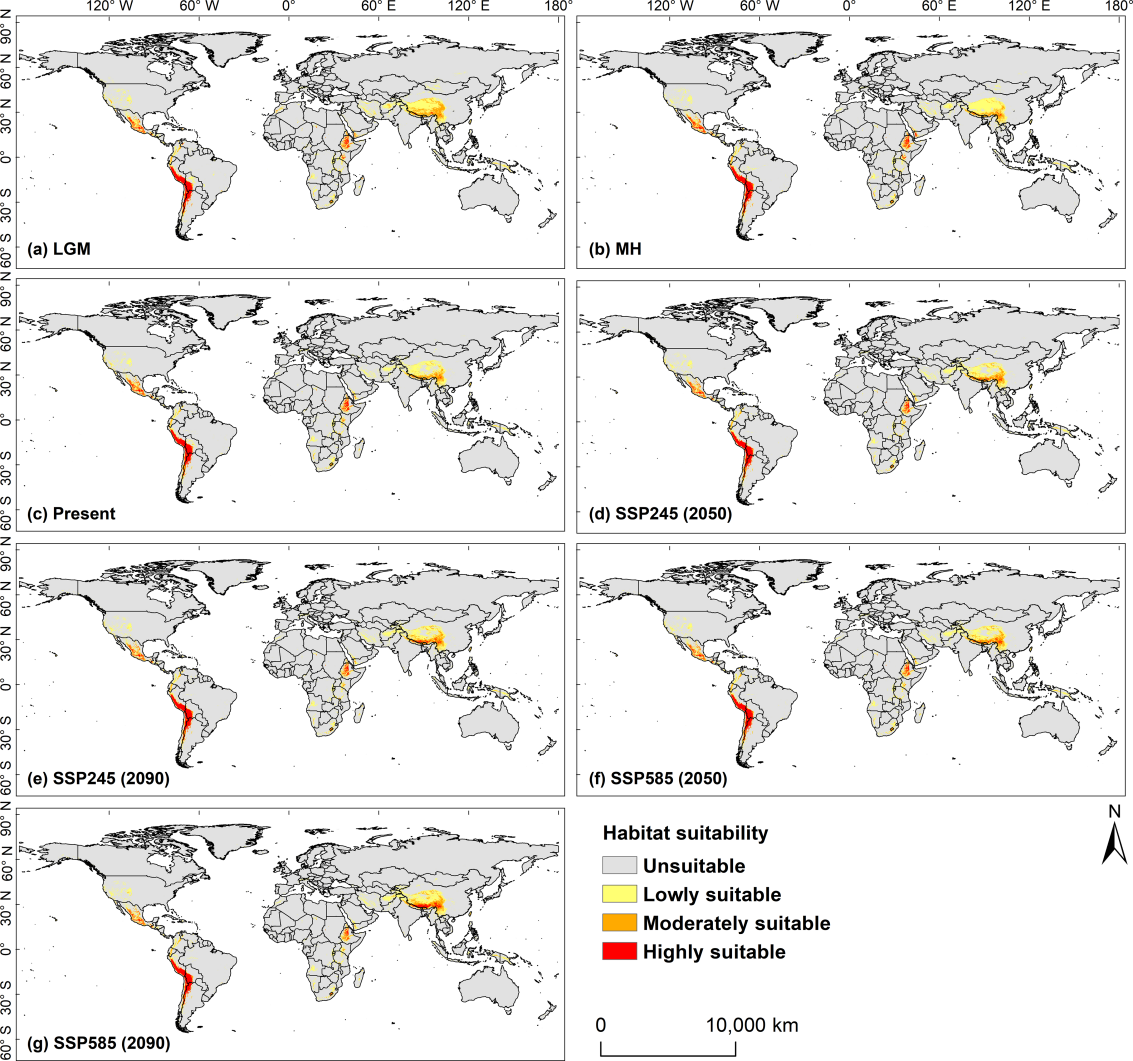
Global potential suitable habitat for *Lepidium meyenii* across different time periods, predicted using the random forest (RF) model. Abbreviations: LGM = Last Glacial Maximum, MH = Mid-Holocene. Habitat suitability classes: unsuitable, 0–0.2; low suitability, 0.2–0.4; moderate suitability, 0.4–0.6; high suitability, 0.6–1. **(a, b)** past projections under paleoclimatic conditions (LGM and MH); **(c)** Present; **(d, e)** future projections under the SSP245 scenario (2050 and 2090); **(f, g)** future projections under the SSP585 scenario (2050 and 2090).

The simulation results indicated that highly suitable habitats of *L. meyenii* were primarily concentrated in the Andes Mountains of western South America, closely corresponding to the current sampling locations. Moderately suitable habitats were mainly distributed across the Tibetan Plateau in Asia, the Mexican Plateau in North America, and the Ethiopian Highlands in eastern Africa, whereas lowly suitable habitats were more broadly and discontinuously distributed (**Figure 5**).

The responses of suitable habitats to climatic changes differed markedly among regions. Throughout the examined periods, the suitable habitats in the Andes Mountains, the Mexican Plateau, and the Ethiopian Highlands remained relatively stable (**Figure 5**). In contrast, substantial changes were observed on the Tibetan Plateau. During the LGM and the MH, suitable habitats were dominated by moderately and lowly suitable areas. At present and under the SSP245 scenario in 2050, highly suitable habitats began to emerge in the southern Tibetan Plateau. Under the SSP585 scenario in 2050 and under both SSP245 and SSP585 scenarios in 2090, highly suitable habitats expanded further along the southern margin of the Tibetan Plateau, reaching their greatest extent under SSP585 in 2090.

The total area of potential suitable habitat exhibited pronounced temporal dynamics (**Table 1**). During the LGM, the total suitable habitat area reached 1075.59 × 10^4^ km^2^, representing the largest extent among all periods examined, including 166.54 × 10^4^ km^2^ of highly suitable habitat (**Table 1**). From the LGM to the MH, the area of moderately suitable habitat decreased from 228.62 × 10^4^ km^2^ to 173.11 × 10^4^ km^2^, representing a decline of 24.28%, while the total suitable habitat area decreased by 7.71% (**Table 1**). From the MH to the present, both highly and lowly suitable habitats declined by ~9%, accompanied by a further reduction of 7.76% in total suitable habitat area (**Table 1**).

**Table 1:** The area (10^4^ km^2^) of potential suitable habitats of *Lepidium meyenii*.

Period	Lowly suitable		Moderately suitable		Highly suitable		Total	
	Area	Change	Area	Change	Area	Change	Area	Change
LGM	680.43	NA	228.62	NA	166.54	NA	1075.5	NA
MH	646.83	-4.94%	173.11	-24.28%	172.67	3.72%	992.61	-7.71%
Present	583.67	-9.76%	174.71	0.92%	157.18	-8.98%	915.56	-7.76%
SSP245 (2050)	549.29	-5.89%	186.58	6.81%	160.07	1.84%	895.94	-2.14%
SSP585 (2050)	550.23	-5.74%	188.25	7.73%	159.43	1.40%	897.91	-1.94%
SSP245 (2090)	552.12	0.51%	189.61	1.61%	163.34	2.01%	905.07	1.00%
SSP585 (2090)	591.45	7.49%	192.65	2.34%	174.37	9.37%	958.47	6.74%

LGM, Last Glacial Maximum; MH, Mid-Holocene; NA, not applicable.

Under future climate scenarios, suitable habitat areas are projected to follow different trajectories (**Table 1**). Under SSP245, the total suitable habitat area is projected to decrease from 915.56 × 10^4^ km^2^ at present to 895.94 × 10^4^ km^2^ by 2050, representing a reduction of 2.14% (**Table 1**). In contrast, highly suitable habitat is projected to increase from 157.18 × 10^4^ km^2^ to 160.07 × 10^4^ km^2^, while moderately suitable habitat is projected to increase from 174.71 × 10^4^ km^2^ to 186.58 × 10^4^ km^2^ (**Table 1**). Between 2050 and 2090, all suitability classes are projected to show slight increases in area (**Table 1**).

Under SSP585, the total suitable habitat area is projected to decline to 897.91 × 10^4^ km^2^ by 2050, whereas moderately and highly suitable habitats are expected to increase by 7.73% and 2.01%, respectively, relative to the present (**Table 1**). From 2050 to 2090, the total suitable habitat area is projected to increase to 958.47 × 10^4^ km^2^, with lowly, moderately, and highly suitable habitats increasing by 7.49%, 2.34%, and 9.37%, respectively (**Table 1**).

### 3.4 Changes in potential habitat suitability since the LGM

**Figure 6:**
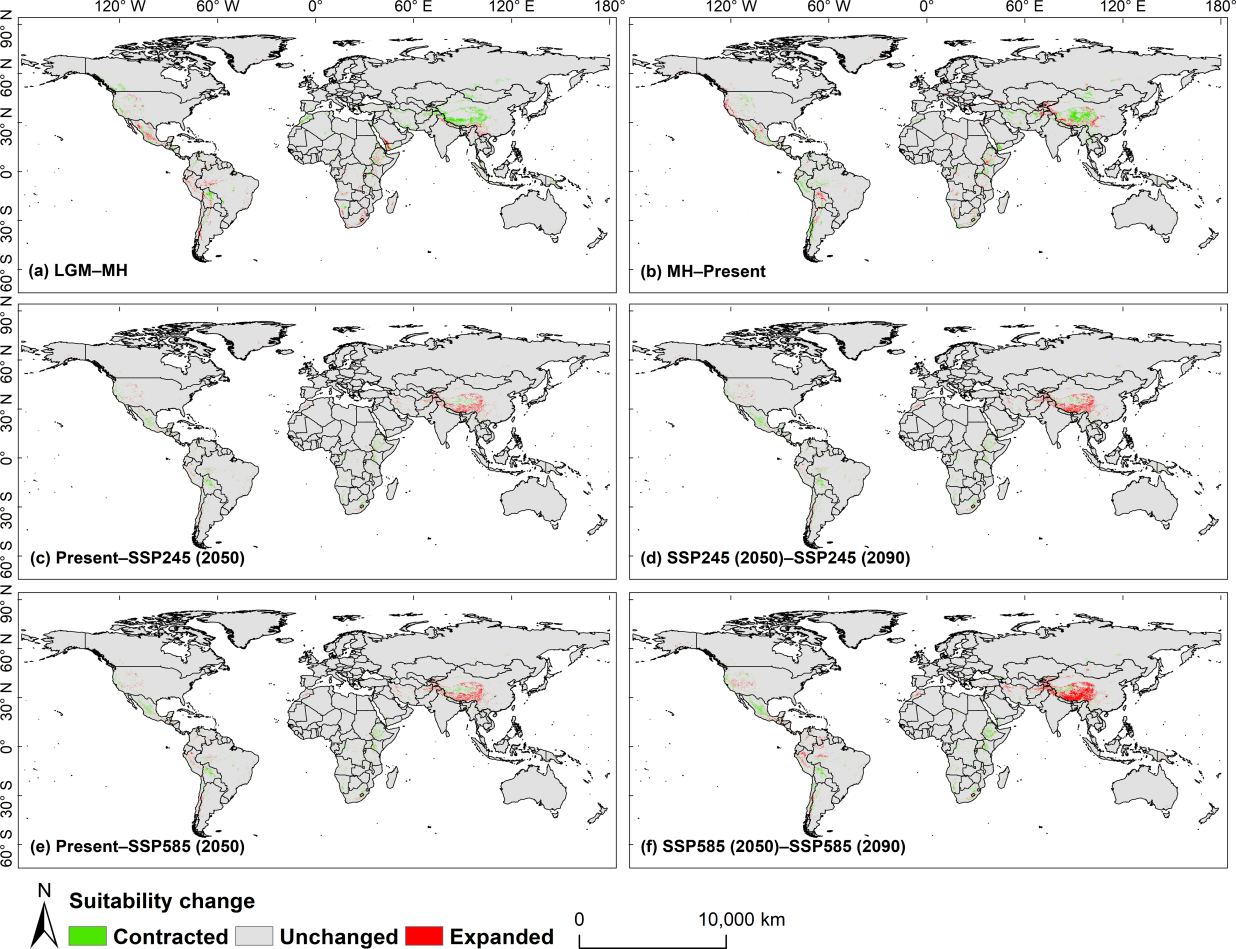
Temporal changes in global potential suitable habitats of *Lepidium meyenii* under various climate scenarios. LGM, Last Glacial Maximum; MH, Mid-Holocene. **(a, b)** changes between past climatic periods; **(c, d)** changes from the present to and between future periods under the SSP245 scenario; **(e, f)** changes from the present to and between future periods under the SSP585 scenario.

Overall, the expansion and contraction of the suitable habitats of *L. meyenii* were concentrated primarily on the Tibetan Plateau, whereas changes in habitat suitability were relatively limited in the Andes, the Mexican Plateau, and the Ethiopian Plateau (**Figure 6**; **Table 2**).

**Table 2:** Temporal changes in potential suitable habitats (10^4^ km^2^) of *Lepidium meyenii*.

Period	Unchanged	Contracted	Expanded
LGM–MH	13057.13	254.43	131.34
MH–Present	13065.61	241.49	135.79
Present–SSP245 (2050)	13249.89	97.53	95.48
SSP245 (2050)–SSP245 (2090)	13215.50	113.52	113.88
Present–SSP585 (2050)	13205.05	111.12	126.72
SSP585 (2050)–SSP585 (2090)	13066.92	145.62	230.36

LGM, Last Glacial Maximum; MH, Mid-Holocene.

During the LGM–MH period, the area of habitat expansion was estimated at 131.34 × 10^4^ km^2^ and was scattered across different regions, whereas the area of habitat contraction reached 254.43 × 10^4^ km^2^ and was mainly concentrated on the Tibetan Plateau. During the MH–Present period, the area of habitat expansion increased slightly to 135.79 × 10^4^ km^2^, with part of the expansion occurring along the southern margin of the Tibetan Plateau. In contrast, the contraction area was estimated at 241.49 × 10^4^ km^2^ and remained largely concentrated on the Tibetan Plateau.

Under future climate scenarios, habitat expansion of *L. meyenii* is projected to occur mainly along the southern margin of the Tibetan Plateau, whereas habitat contraction is projected to be scattered across the northwestern Tibetan Plateau, the Mexican Plateau, the northern Andes, and the Ethiopian Plateau. Under the SSP245 scenario, changes in suitable habitats are projected to be relatively moderate. By 2050, the contraction and expansion areas are expected to reach 97.53 × 10^4^ km^2^ and 95.48 × 10^4^ km^2^, respectively. By 2090, these areas are projected to increase to 113.52 × 10^4^ km^2^ and 113.88 × 10^4^ km^2^, respectively. In contrast, more significant changes are projected under the SSP585 scenario. By 2050, the contraction and expansion areas are expected to reach 111.12 × 10^4^ km^2^ and 126.72 × 10^4^ km^2^, respectively. By 2090, both areas are projected to increase further, with the contraction area reaching 145.62 × 10^4^ km^2^ and the expansion area reaching 230.36 × 10^4^ km^2^.

### 3.5 Temporal centroid migration of moderately to highly suitable areas for *L. meyenii*

**Figure 7:**
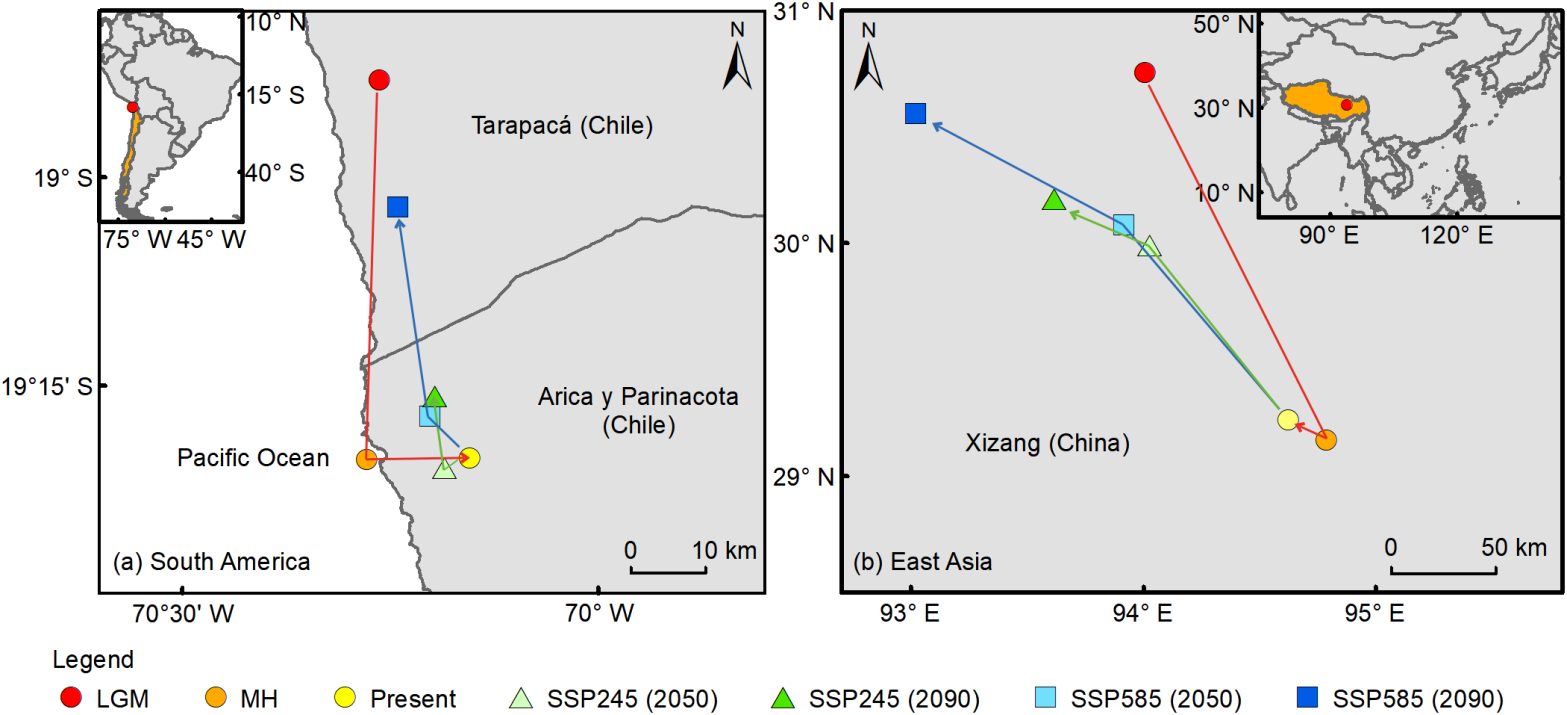
Temporal changes in the migration trends of the geometric centers of potential suitable habitats for *Lepidium meyenii* under various climate scenarios in South America **(a)** and East Asia **(b)**. LGM, Last Glacial Maximum, MH, Mid-Holocene. The lines represent the migration pathways of the centroids, and indicate centroid movement over time: orange—from the past to the present; green—from the present to the future under the SSP245 scenario; blue—from the present to the future under the SSP585 scenario.

Overall, the centroid of suitable habitats for *L. meyenii* exhibited greater spatial displacement in East Asia than in South America. In South America, the centroid of suitable habitats generally exhibited a southward-to-northward migration trend over time (**Figure 7a**; **Table 3**). From the LGM to the MH, the centroid shifted southward by 50.69 km, moving from 18.88° S, 70.26° W to 19.34° S, 70.28° W. By the present, the centroid had moved a further 12.99 km eastward to 19.34° S, 70.15° W. Under the SSP245 scenario, the centroid is projected to shift 3.39 km southwestward to 19.35° S, 70.18° W by 2050, and then move 9.60 km northward to 19.26° S, 70.20° W by 2090. Under the SSP585 scenario, the centroid is projected to migrate 7.50 km northwestward to 19.29° S, 70.20° W by 2050, followed by a further northward shift of 28.29 km to 19.04° S, 70.24° W by 2090.

**Table 3:** Changes in centroid positions of potential suitable habitats and migration distances across different time periods.

Period	South America		East Asia	
	Coordinates	Distance (km)	Coordinates	Distance (km)
LGM	18.88° S, 70.26° W	NA	30.73° N, 94.01° E	NA
MH	19.34° S, 70.28° W	50.69	29.15° N, 94.79° E	191.01
Present	19.34° S, 70.15° W	12.99	29.24° N, 94.62° E	18.66
SSP245 (2050)	19.35° S, 70.18° W	3.39	29.99° N, 94.03° E	101.38
SSP585 (2050)	19.29° S, 70.20° W	7.50	30.08° N, 93.92° E	115.42
SSP245 (2090)	19.26° S, 70.20° W	9.60	30.19° N, 93.62° E	45.38
SSP585 (2090)	19.04° S, 70.24° W	28.29	30.56° N, 93.02° E	101.31

NA, not applicable.

In East Asia, the centroid of suitable habitats generally followed a southeastward-to-northwestward migration trend (**Figure 7b**; **Table 3**). From the LGM to the MH, the centroid shifted 191.01 km southeastward, from 30.73° N, 94.01° E to 29.15° N, 94.79° E. By the present, it had migrated 18.66 km northwestward to 29.24° N, 94.62° E. Under the SSP245 scenario, the centroid is projected to continue migrating northwestward by 101.38 km, reaching 29.99° N, 94.03° E by 2050, and then moving a further 45.38 km to 30.19° N, 93.62° E by 2090. Under the SSP585 scenario, the centroid is projected to shift to 30.08° N, 93.92° E by 2050, representing a migration distance of 115.42 km, and then continue northwestward to 30.56° N, 93.02° E by 2090, with an additional migration distance of 101.31 km.

## 4 Discussion

### 4.1 The evaluation of model performance

We optimized the MaxEnt model with “ENMeval” and compared it with other models available in “biomod2”. The results indicate that the RF model outperforms others in simulating the potential suitable habitats of *L. meyenii* through time. The optimized MaxEnt model shows a significant improvement over the default parameters, with an increase of 0.005 in AUC and 0.006 in TSS, though its accuracy remained lower than that of the RF model.

As an ensemble learning method based on the classification and regression trees (CART) model, RF is widely used in ecology and biogeography due to its stability, insensitivity to parameter tuning, and ability to maintain high accuracy even with small sample sizes (Fox et al., 2017; Luan et al., 2020). While the RF model showed superior performance here, the optimal model may vary across species and at different modeling scales. For instance, community-level models (CLMs) are more appropriate when the aim is to characterize community composition patterns across multiple species, rather than focusing on individual species (Shabani et al., 2016; Nieto‐Lugilde et al., 2018). Therefore, we argue that model selection should be guided by aims and informed by quantitative evaluation, rather than assuming the existence of a universally optimal model.

### 4.2 Key environmental variables and potential distribution in the present day

The most critical factors that influence the potential suitable habitat distribution of *L. meyenii* are elevation, temperature annual range (bio7), mean diurnal range (bio2), and slope, while precipitation plays a relatively minor role (**Figures 3**, **4**). These key environmental factors provide a mechanistic basis for understanding the adaptation of *L. meyenii* across both native and introduced ranges.

The Andes Mountains in western South America represent the main natural distribution area of *L. meyenii*, while Yunnan in China accounts for the largest area of cultivated introduction (Li et al., 2018). Both regions are influenced by monsoon systems and alpine topography, resulting in broadly similar climatic characteristics that are moderately favorable for the growth of *L. meyenii* (Espinoza et al., 2020; Turner and Annamalai, 2012). This distribution pattern indicates that *L. meyenii* is well adapted to alpine environments. This species possesses specific genes and associated molecular mechanisms that enable it to cope with low temperatures and intense ultraviolet radiation, providing important molecular evidence for its adaptation to plateau environments (Zhang et al., 2016). From a physiological-ecological perspective, the pronounced diurnal temperature range and relatively stable annual temperature regime of high-altitude regions can enhance daytime photosynthetic efficiency while reducing nighttime respiratory losses, thereby promoting organic matter accumulation (Collatz et al., 2000). Our results further support these molecular and physiological mechanisms from the perspective of species distribution patterns, and also provide a climatic and topographic reference for the introduction and cultivation of *L. meyenii* in other regions. Meanwhile, species distribution is influenced not only by climate and topography, but also by other environmental factors such as solar radiation, soil, and water availability (Boulangeat et al., 2012). Future studies should therefore incorporate a broader range of environmental variables to better understand the mechanisms underlying the distribution and adaptation of *L. meyenii*.

### 4.3 Changes in potential suitable habitat from past to present

During the LGM–MH transition, projected contraction of potential suitable habitats for *L. meyenii* was concentrated mainly in central South America and the Tibetan Plateau (**Figure 6a**), while habitat expansion was comparatively limited. This pattern may have been driven by climatic warming and shifts in regional monsoon dynamics during the LGM–MH period.

During the LGM, cooling in the Northern Hemisphere caused a southward shift of the Intertropical Convergence Zone (ITCZ). In contrast, during the MH, the ITCZ shifted northward, strengthening monsoon activity in northern South America while weakening it in central and southern regions (Berman et al., 2018). Consequently, the model projected a substantial increase in potential suitable habitats in northern South America, whereas habitat suitability declined more markedly in central South America (**Figure 6a**).

In East Asia, compared with the LGM, the generally warmer climate and orbital-scale climatic changes during the MH may have altered environmental conditions in alpine regions, thereby influencing the distribution of potential suitable habitats for *L. meyenii* (D'Agostino et al., 2019). At the regional scale, the MH was characterized by a strengthened East Asian summer monsoon and a weakened winter monsoon, which may have reduced the mean diurnal temperature range (bio2) (Zhao and Yu, 2012). These climatic changes may together have contributed to the overall contraction of projected potential suitable habitats across the Tibetan Plateau (**Figure 6a**).

From the MH to the present, the contraction of potential suitable habitats for *L. meyenii* occurred primarily across the Tibetan Plateau and northern South America, whereas habitat expansion was relatively limited and restricted to a few areas along the southern margin of the Tibetan Plateau (**Figure 6b**). This contraction may be closely associated with elevation-dependent warming (EDW), a phenomenon in which warming rates are greater at higher elevations than at lower elevations as a result of increasing greenhouse gas concentrations and associated land-atmosphere feedback (Mountain Research Initiative EDW Working Group, 2015). Both the Tibetan Plateau and the Andes Mountains have been identified as regions experiencing pronounced EDW, which has accelerated climatic change in alpine ecosystems and altered vegetation distribution patterns (Mountain Research Initiative EDW Working Group, 2015). For cold-adapted alpine species such as *L. meyenii*, continued warming may reduce the extent of climatically suitable environments, leading to habitat contraction and potentially exerting negative impacts on regional biodiversity (Toledo et al., 2022).

When comparing the LGM–MH and MH–present periods, the primary areas with contraction and expansion for *L. meyenii* show opposite patterns, particularly in the northern-central South America. Similarly, many high-altitude, cold-adapted plant species have shown comparable changes in projected suitable habitat during these two periods (Xiao et al., 2022).

We conclude that natural environmental factors primarily drove changes in plant distribution during the LGM–MH, while human activities have increasingly influenced distribution during the MH–present period, particularly under the EDW effect in high-altitude regions. Therefore, future studies should integrate human activities as an environmental variable in SDM to better understand their impact on shifts in species potential suitable habitats.

### 4.4 Changes in potential suitable habitat from the present to the future

The future spatial dynamics of potential suitable habitats for *L. meyenii* exhibit a broadly consistent pattern. Habitat contraction is projected to occur primarily in the northwestern Tibetan Plateau, the Mexican Plateau of North America, and central South America, whereas the most pronounced habitat expansion is expected in the southern regions of the Tibetan Plateau.

On the Mexican Plateau, although a certain extent of potential suitable habitat is projected to persist under current climatic conditions, future climate change may lead to further habitat contraction (**Figures 5c**, **6c–f**). The Mexican Plateau has a mean elevation of ~2, 250 m, which is generally lower than the typical elevational range of *L. meyenii* in its native Andean habitats (Whiteman et al., 2000). Previous studies have suggested that future climate warming in Mexico will be accompanied by increasing temperatures and declining precipitation, driving upslope shifts in the climatic niches of many montane plant species (Gómez-Pineda et al., 2020). As a cold-adapted alpine herb, *L. meyenii* may undergo a similar upward shift in its climatic niche. However, the availability of higher-elevation environments for *L. meyenii* on the Mexican Plateau may be relatively limited. Consequently, the extent of cool environments suitable for the growth of *L. meyenii* may progressively decline under future warming, leading to a contraction of its potential suitable habitats.

Within the native South American range, the spatial configuration of potential suitable habitats exhibits a slight northward shift, as reflected by the progressive northward migration of the habitat centroid from the present to future periods (**Figure 7a**). However, the overall extent of suitable habitat changes little. This suggests that the potential range of *L. meyenii* in its native range is likely to remain relatively stable under projected future climate scenarios.

In the introduced range in southwestern China, the potential suitable habitats of *L. meyenii* are projected to contract in the northwestern Tibetan Plateau, while expansion is observed in the southern part. This north-south differentiation may be influenced by regional climatic conditions. The southern Tibetan Plateau experiences stronger warm and humid airflow, with relatively low bio7 and bio2, which may facilitate the expansion of potential suitable habitats (Schiemann et al., 2009; Zhou et al., 2018). In contrast, northern inland areas such as the Tarim Basin are colder and drier, with higher bio7, which may contribute to the contraction of potential suitable habitats (Pan et al., 2020; Schiemann et al., 2009).

In addition, this north-south pattern may also be associated with the topographic characteristics of the Tibetan Plateau and EDW. The southern Tibetan Plateau is generally higher in elevation than the north, and under global warming, some species may shift toward higher elevations (Spicer et al., 2021). Previous studies indicate that under future climate scenarios, the Tibetan Plateau is expected to experience substantial warming, with rates increasing with elevation, reflecting a pronounced EDW pattern (You et al., 2020; Zhou et al., 2023). Therefore, the expansion of potential suitable habitats in the southern high-elevation regions may be driven not only by enhanced warm and humid airflows but also by improved environmental conditions resulting from warming at higher elevations. Conversely, the contraction in the northern regions reflects the continued influence of unfavorable climatic conditions. Moreover, previous studies have suggested that global warming may induce range shifts in some alpine herbaceous species on the Tibetan Plateau, which is similar with this study (e.g., Fan and Bai, 2021). Therefore, we suggest that the southern margin of the Tibetan Plateau may become a more important area of potential suitable habitat for *L. meyenii* under projected future climate conditions.

It should be noted that SDMs reflect spatial changes in environmental suitability, while actual distribution patterns may also be shaped by dispersal limitations, biotic interactions, and other ecological processes (Moonlight et al., 2020). Therefore, future studies should integrate process-based models with multi-source data to improve the explanatory power and predictive reliability of species distribution dynamics.

Our results indicate that the southern border of the Tibetan Plateau may become a more important region of potential suitable habitats for *L. meyenii* in the future. These regions may warrant further evaluation for potential cultivation from a climatic perspective, rather than restricting cultivation only to Yunnan. However, actual cultivation feasibility would also depend on additional environmental and socio-economic factors not considered in this study, including soil properties, hydrological conditions, land-use patterns, and agricultural management. In addition, climate change may lead to niche shifts and alter interspecific interactions. Therefore, future cultivation and management could integrate both socio-economic and environmental factors to ensure sustainable utilization.

### 4.5 Caveats

Several caveats of this study should be acknowledged. First, our analyses relied on 86 occurrence records of *L. meyenii*. As an alpine species with a narrow range and limited field documentation, its available occurrence data are intrinsically sparse, a common constraint in studies of rare or data−deficient taxa. Although datasets of comparable size have been widely and effectively used in SDMs such as MaxEnt and RF (Moudrý and Šímová, 2012; Lan et al., 2022), small sample sizes may not fully capture the realized environmental space, potentially leading to under− or overestimation of suitable habitat in some regions. Second, model predictions are subject to uncertainties in the underlying climate data. The environmental predictors were derived from gridded datasets that may not resolve fine−scale microclimatic heterogeneity in complex mountain terrain, and, although we reduced inter−model variability by ensemble−averaging multiple GCMs, we did not explicitly quantify inter−GCM uncertainty. As a result, the projected distributions may still be influenced by model-dependent biases. Future work incorporating additional field-based occurrence records, higher−resolution climate layers, and formal assessment of GCM-related uncertainty would improve the robustness and reliability of the projections.

## 5 Conclusions

Using the species distribution data of *L. meyenii* alongside multiple environmental variables, we evaluated 11 species distribution models and ultimately selected the RF model as the optimal approach. This model allowed us to pinpoint the key environmental factors that influence the distribution of *L. meyenii* and to project its potential suitable habitats through time. We find that the distribution of *L. meyenii* is primarily influenced by elevation and temperature rather than precipitation. Specifically, *L. meyenii* favors high elevations, a low bio7, and a high bio2.

Driven by climate change, the total area of potential suitable habitat for *L. meyenii* exhibited an overall pattern of initial decline followed by expansion from the past to the future. Historically, highly suitable habitats were concentrated mainly in western South America, whereas moderately suitable habitats were primarily distributed across the Tibetan Plateau, the Mexican Plateau, and the Ethiopian Highlands. From the LGM to the present, the potential suitable habitat of *L. meyenii* generally contracted under the combined influence of global climatic changes and regional climatic dynamics, with the major habitat loss occurring on the Tibetan Plateau. However, this trend is projected to reverse under future climate scenarios. The Andes Mountains of South America are expected to remain an important natural habitat for *L. meyenii*. With increasing greenhouse gas emission scenarios, potential suitable habitats are projected to contract in the northwestern Tibetan Plateau and the Mexican Plateau. In contrast, highly suitable habitats in the southern Tibetan Plateau are expected to expand further, a pattern that may be associated with ongoing climate warming and EDW. These results suggest that the southern Tibetan Plateau, together with the Andes Mountains, may become important regions of potential suitable habitat for *L. meyenii* in the future, with potential implications for its sustainable utilization. Centroid analysis showed that, in South America, the centroid of suitable habitats for *L. meyenii* exhibited a southward-to-northward migration trend through time. In East Asia, the centroid generally followed a southeastward-to-northwestward migration trajectory during the historical period.

## Data Availability

Publicly available datasets were analyzed in this study. This data can be found here: https://doi.org/10.15468/dl.rvuepx http://worldclim.org.
