# Supplementary material for: Unveiling the potential geographic range of Lepidium meyenii (Maca) and its climate-driven shifts in a changing climate
Source: Front Plant Sci. 2026 Jul 15;17:1839669. doi: 10.3389/fpls.2026.1839669 (PMC13415769; doi:10.3389/fpls.2026.1839669)
Supplement: Supplementary file 1 [file DataSheet1.pdf]

Supplementary Material

Supplementary Figure S1. Pearson correlation matrix of 22 environmental variables. Abbreviations: bio1 = annual mean temperature, bio2 = mean diurnal range, bio3 = isothermality, bio4 = temperature seasonality, bio5 = max temperature of warmest month, bio6 = min temperature of coldest month, bio7 = temperature annual range, bio8 = mean temperature of wettest quarter, bio9 = mean temperature of driest quarter, bio 10 = mean temperature of warmest quarter, bio11 = mean temperature of coldest quarter, bio12 = annual precipitation, bio13 = precipitation of wettest month, bio14 = precipitation of driest month, bio15 = precipitation seasonality, bio16 = precipitation of wettest quarter, bio17 = precipitation of driest quarter, bio18 = precipitation of warmest quarter, bio19 = precipitation of coldest quarter, aspect = orientation of surface direction, elevation = height of a location above sea level, slope = ratio of vertical change to horizontal distance.

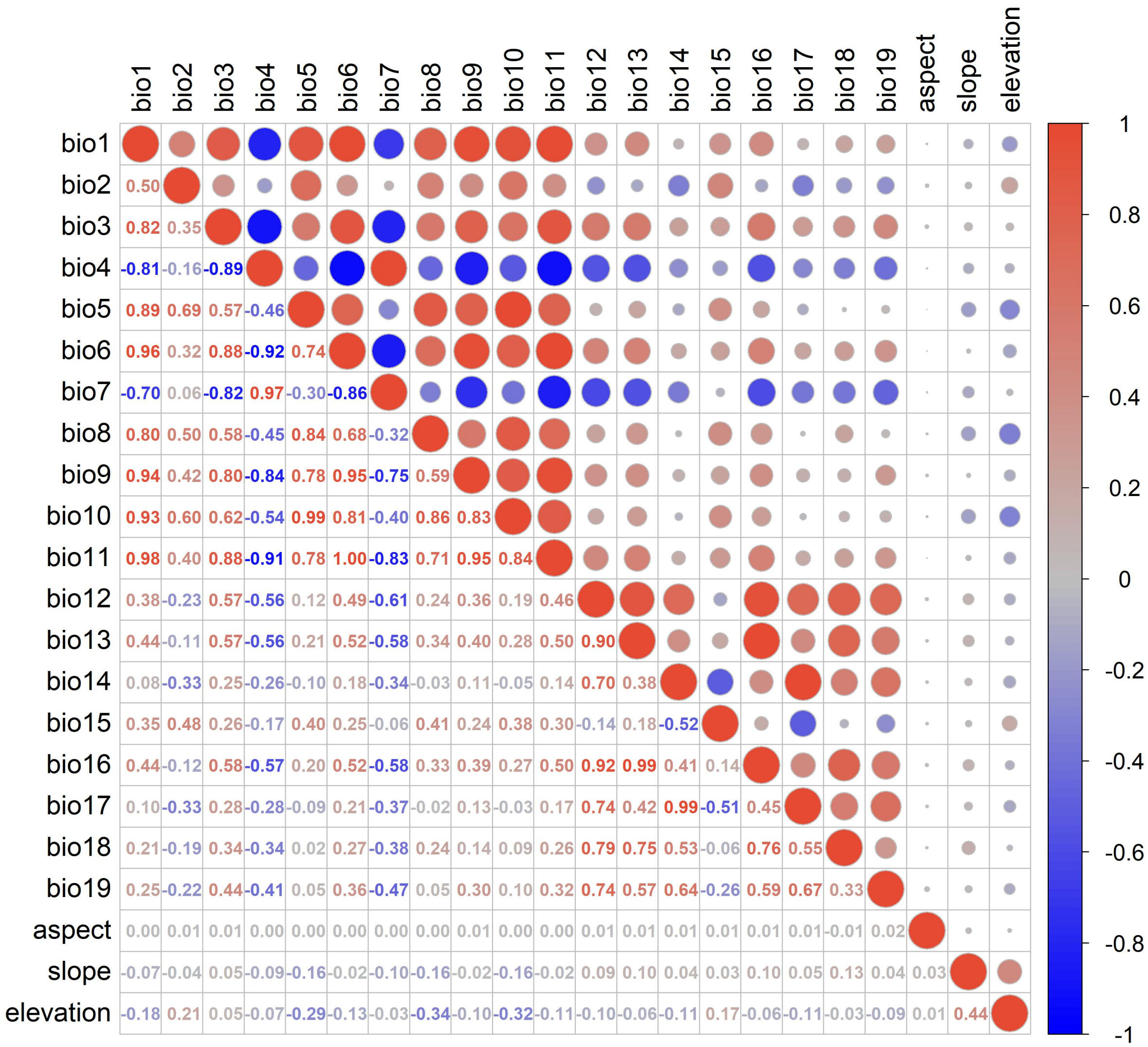

Supplementary Figure S2. Spearson correlation matrix of 22 environmental variables. The full names of all abbreviations are provided in the legend of Supplementary Figure 1.

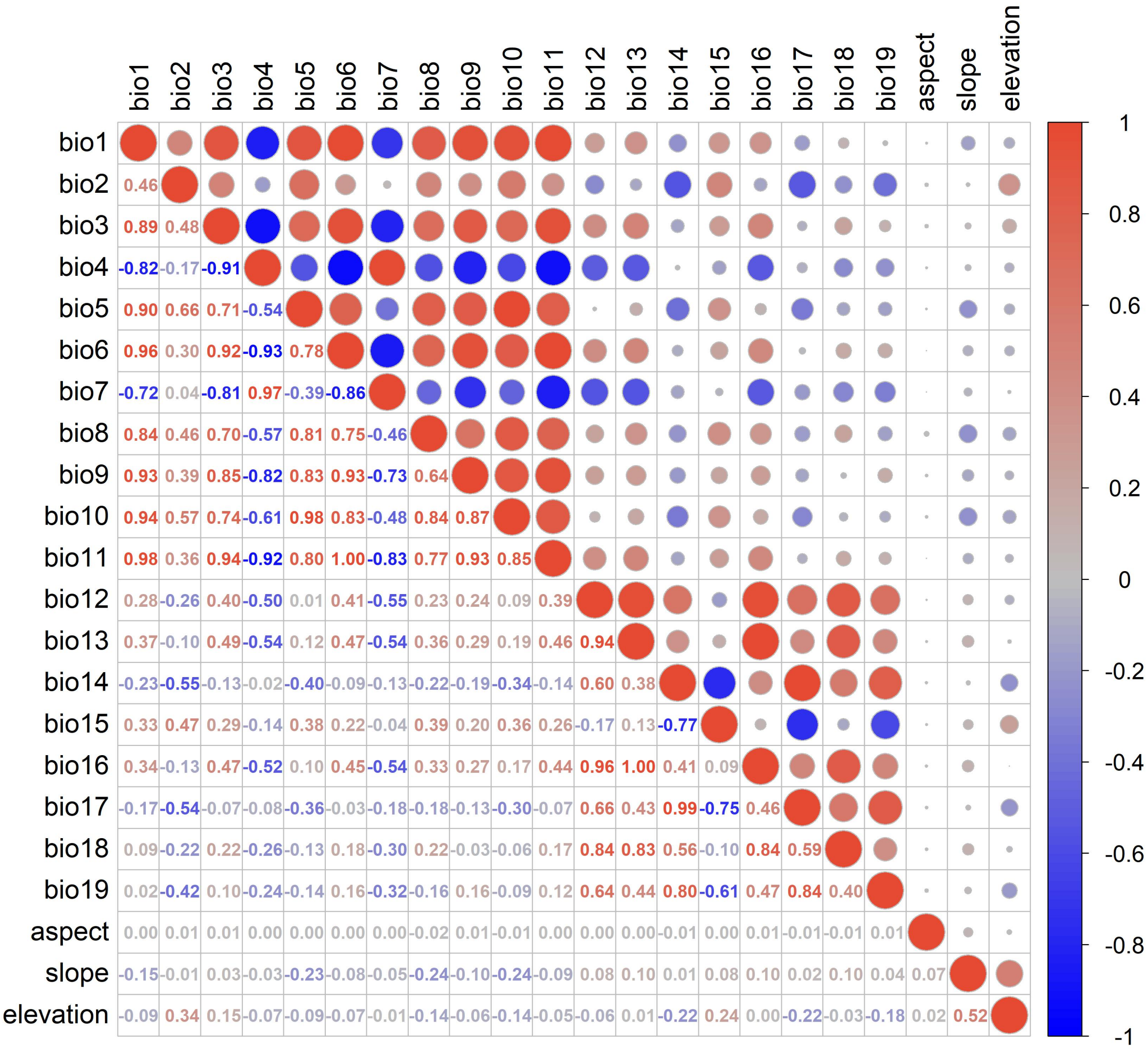

Supplementary Table S1. Details of the 22 environmental variables used for evaluation

Variable	Description	Unit
bio1	Annual Mean Temperature	°C
bio2	Mean Diurnal Range (Mean of monthly (max temp–min temp))	°C
bio3	Isothermality (bio2/bio7) (×100)	/
bio4	Temperature Seasonality (standard deviation ×100)	/

bio5	Max Temperature of Warmest Month	°C
bio6	Min Temperature of Coldest Month	°C
bio7	Temperature Annual Range (bio5–bio6)/°C	°C
bio8	Mean Temperature of Wettest Quarter	°C
bio9	Mean Temperature of Driest Quarter	°C
bio10	Mean Temperature of Warmest Quarter	°C
bio11	Mean Temperature of Coldest Month	°C
bio12	Annual Precipitation	mm
bio13	Precipitation of Wettest Month	mm
bio14	Precipitation of Driest Month	mm
bio15	Precipitation Seasonality (Coefficient of Variation)	/
bio16	Precipitation of Wettest Quarter	mm
bio17	Precipitation of Driest Quarter	mm
bio18	Precipitation of Warmest Quarter	mm
bio19	Precipitation of Coldest Quarter	mm
aspect	Orientation of Surface Direction	/
elevation	Height of a Location Above Sea Level	m
slope	Ratio of Vertical Change to Horizontal Distance	°

Supplementary Table S2. The result of variance inflation factor (VIF)

**Variables**	**VIF value**
bio1	9.79
bio2	4.75
bio7	6.42
bio12	3.30
bio14	3.16
bio15	1.97
aspect	1.00
elevation	2.53
slope	9.79

Supplementary Table S3. Optimization results of the MaxEnt model based on AICc. Lower AICc values indicate a better-fitting model. The optimal parameter combination is highlighted in bold.

Feature combination (FC)	Regulated frequency multiplier (RM)	AICc
H	0.5	1935.56
L	0.5	1835.42
LQ	0.5	1812.01
LQH	0.5	1896.42
LQHP	0.5	1912.44
LQHPT	0.5	1840.71
H	1	1884.09
L	1	1835.59
**LQ**	**1**	**1818.93**
LQH	1	1832.85
LQHP	1	1845.89
LQHPT	1	1839.54
H	1.5	1892.58
L	1.5	1836.18
LQ	1.5	1824.28
LQH	1.5	1832.27
LQHP	1.5	1840.21
LQHPT	1.5	1843.70
H	2	1905.32
L	2	1839.21
LQ	2	1831.36
LQH	2	1833.60
LQHP	2	1834.70
LQHPT	2	1834.59
H	2.5	1908.02
L	2.5	1842.22
LQ	2.5	1833.15
LQH	2.5	1832.97
LQHP	2.5	1839.27
LQHPT	2.5	1839.35
H	3	1916.03
L	3	1845.29
LQ	3	1836.95
LQH	3	1836.89

LQHP	**3**	1846.02
LQHPT	3	1842.97
H	3.5	1929.01
L	3.5	1848.35
LQ	3.5	1840.53
LQH	3.5	1840.62
LQHP	3.5	1849.59
LQHPT	3.5	1849.58
H	4	1931.49
L	4	1851.41
LQ	4	1844.31
LQH	4	1844.72
LQHP	4	1855.64
LQHPT	4	1855.47
H	4.5	1942.56
L	4.5	1854.47
LQ	4.5	1848.09
LQH	4.5	1848.29
LQHP	4.5	1861.56
LQHPT	4.5	1861.56
H	5	1948.83
L	5	1857.52
LQ	5	1851.99
LQH	5	1852.08
LQHP	5	1864.12
LQHPT	5	1864.12
H	5.5	1963.63
L	5.5	1860.54
LQ	5.5	1856.06
LQH	5.5	1855.75
LQHP	5.5	1869.14
LQHPT	5.5	1869.08
H	6	1971.92
L	6	1863.55
LQ	6	1859.41
LQH	6	1859.50
LQHP	6	1874.08
